# Dose-dependent Effect of Statin Therapy on Circulating CXCL12 Levels in Patients with Hyperlipidemia

**DOI:** 10.1186/2001-1326-1-23

**Published:** 2012-10-06

**Authors:** Will Camnitz, Marie D Burdick, Robert M Strieter, Borna Mehrad, Ellen C Keeley

**Affiliations:** 1 From the Department of Medicine, Division of Cardiology, University of Virginia, PO Box 800158, Charlottesville, Virginia, USA; 2 From the Department of Medicine, Division of Pulmonary Critical Care, University of Virginia, PO Box 800546, Charlottesville, Virginia, USA

**Keywords:** CXCL12, Chemokine, Statin

## Abstract

**Background:**

HMG-CoA reductase inhibitors (statins) have pleiotropic effects that are independent of cholesterol-lowering, including a dose-dependent effect on angiogenesis. Angiogenesis plays a critical role both in vascularization of the chronically ischemic myocardium and in stabilization of atherosclerotic plaques. Chemokines, a family of structurally-related cytokine molecules, exert diverse biological functions including control of angiogenesis. The effect of statin therapy on angiogenic and angiostatic chemokines has not been evaluated extensively. We sought to test the hypothesis that, in subjects with hyperlipidemia, statin therapy influences plasma levels of angiogenic and angiostatic chemokines in a dose-dependent manner.

**Methods:**

We prospectively collected demographic, angiographic and laboratory data from subjects with a history of hyperlipidemia who were either untreated or on statin therapy. A peripheral blood sample was obtained for measurement of plasma angiogenic and angiostatic chemokines. Multivariable analysis using logistic regression was performed adjusting for the following variables: age, gender, prior myocardial infarction, and chronic administration of aspirin, clopidogrel, insulin, oral hypoglycemic agents, beta-blockers and calcium channel blockers.

**Results:**

168 patients on statin therapy (48 on low-dose, defined as <10mg atorvastatin-equivalent, and 120 on high-dose, defined as ≥10mg atorvastatin-equivalent dose) and 11 subjects from the same database who had a history of hyperlipidemia but who were not on statins were enrolled. There were no significant differences in baseline demographics, co-morbidities, lipid panels, other medications, or angiographic data between the groups. The angiogenic chemokines CXCL1 and CXCL12 levels were significantly different across the groups. Median levels of CXCL1 were highest in subjects not on statin therapy. Compared to subjects either not on statin therapy or on low-dose statins, those taking high-dose statins had lower median values of CXCL12 (2316 [2255–11071], vs 2362 [2016–10622], vs 2189 [1968–2705] pg/mL, p=0.042). On multivariate analysis, CXCL12 remained the only factor that was strongly and inversely associated with statin dose at the 95% level (p=0.011).

**Conclusions:**

Compared to no therapy or low-dose statin therapy, treatment with high-doses of HMG-CoA reductase inhibitors is associated with decreased circulating CXCL12 levels in subjects with hyperlipidemia, and CXCL12 is strongly and inversely associated with statin dose. Additional studies are needed to confirm this finding in other cohorts and to determine if high-dose statins affect angiogenesis in patients.

## Background

HMG-CoA reductase inhibitors are among the most commonly prescribed medications, and have pleiotropic effects that are independent of cholesterol-lowering [1]. Among these, several lines of evidence support a dose-dependent effect of these drugs on angiogenesis; for example, in animal models, inflammation-induced angiogenesis is enhanced with low-dose statin therapy but inhibited at high statin concentrations, and high-dose statin concentration is associated with decreased tumor vascularization in a lung cancer model [2]. Moreover, in vitro studies demonstrate that low-dose statin concentrations are pro-angiogenic by promoting the migration of human endothelial progenitor cells while at high concentrations they are anti-angiogenic by promoting endothelial cell apoptosis [3]. The effects of statins on angiogenesis in humans are likely to be clinically relevant to heart disease since angiogenesis is associated with formation of new blood vessels in chronically ischemic myocardium; in addition, inhibition of angiogenesis may contribute to stabilization of atherosclerotic plaques [4].

Chemokines are a family of structurally-related cytokine molecules with diverse biological functions, including control of angiogenesis. We recently reported that, in subjects with coronary artery disease, the plasma concentration of angiogenic chemokines was independently associated with the presence and extent of angiographically visible coronary collaterals, whereas the concentration of angiostatic chemokines was negatively correlated [5]. The effect of statin therapy on angiogenic and angiostatic chemokines in subjects with coronary artery disease has not been evaluated extensively. Given the high prevalence of statin use in the population, we sought to test the hypothesis that, in subjects with hyperlipidemia, statin therapy influences plasma levels of angiogenic and angiostatic chemokines in a dose-dependent manner.

## Methods

### Patient population

We prospectively collected demographic, angiographic and laboratory data from consecutive subjects with a history of hyperlipidemia undergoing elective coronary angiography at the University of Virginia from October 2007 to August 2008. The study was approved by the Institutional Review Board and all subjects provided informed consent. Patients > 21 years old who were able to provide informed consent were eligible for enrollment. Exclusion criteria were: (1) acute coronary syndrome, (2) active inflammatory, infectious, or malignant disease, and (3) immunosuppressive therapy. Doses of statins were converted to an atoravastatin-equivalent on the basis of their low-density lipoprotein-lowering potential as previously described [6]. Subjects were classified as not on a statin, on low-dose statins (defined as <10mg atorvastatin-equivalent dose which included the following: lovastatin 10mg and 20mg; pravastatin 10mg and 20mg; and simvastatin 5mg and 10mg) or on high-dose statins (defined as ≥10mg atorvastatin-equivalent dose which included the following: atorvastatin 10mg, 20mg, 40mg and 80mg; lovastatin 40mg and 80mg; pravastatin 40mg and 80mg; rosuvastatin 5mg, 10mg, 20mg and 40mg; and simvastatin 20mg, 40mg and 80mg) [6].

### Blood sampling and cytokine analysis

At the time of arterial sheath insertion for coronary angiography and prior to administration of heparin, a blood sample was drawn from the side arm of the sheath and anticoagulated with sodium EDTA, placed on ice, and processed within 30 minutes of retrieval. Platelet-free plasma was aliquoted and frozen at −80°C for subsequent measurement of cytokines by multiplex immunoassay (Luminex, Bio-Rad, Bio-plex 200 system, Hercules, California; Procarta Cytokine Assay kit, Panomics, Inc., Fremont, California). We assayed for the angiogenic chemokines CXCL1, CXCL3, CXCL5, CXCL8, CXCL12 and CCL2, the angiogenic cytokine VEGF, the angiostatic chemokines CXCL9, CXCL10 and CXCL11, and the cytokine interferon-gamma (a potent inducer of angiostatic chemokines).

### Statistical analysis

Baseline characteristics were analyzed using Wilcoxon rank-sum test for continuous and Chi-Square test or Fisher’s Exact test for categorical variables. Statistically significant differences between groups were compared with one-way analysis of variance (ANOVA). Multivariable analysis using logistic regression was performed adjusting for the following variables: age, gender, prior myocardial infarction, and chronic administration of aspirin, clopidogrel, insulin, oral hypoglycemic agents, beta-blockers and calcium channel blockers. Analyses were performed using Stata/ IC version 10.1 (Statacorp) and GraphPad PRISM (version 5.0d). Statistical significance was defined as *p* value of <0.05.

## Results

A total of 168 subjects with a history of hyperlipidemia on chronic statin therapy (low-dose n=48, and high-dose n=120), and 11 subjects with a history of hyperlipidemia not on lipid-lowering therapy were enrolled. There were no significant differences in baseline medications, fasting lipid levels, medical co-morbidities, the presence of coronary artery disease, and the presence of angiographic coronary collaterals between the groups (Table 1). Overall, few subjects had a clinical history of congestive heart failure and there was no difference in its prevalence between the groups: echocardiographic assessment of left ventricular systolic function revealed that both subjects in the no treatment group had mildy depressed function (defined as 45-50%), 2 subjects in the low-dose group had mildy depressed and 1 had moderately depressed (defined as 40-45%) function, and in the high-dose group, 5 had mildly depressed and 4 had moderately depressed function. Subjects taking statins had higher rates of a family history of coronary artery disease (p=0.047) (Table 1).

**Table 1:** Characteristics of Subjects

**Characteristic**	**No statin(n=11)**	**Low dose statin(n=48)**	**High dose statin (n=120)**	**p Value**
Age (years)	65 +/- 14	64 +/- 11	60 +/- 12	0.489
Male gender	8 (73%)	31 (65%)	85 (71%)	0.707
Race				
Caucasian	11 (100%)	41 (85%)	106 (88%)	0.398
African-American	0 (0%)	7 (15%)	14 (12%)	0.398
Hypertension*	7 (64%)	42 (88%)	104 (87%)	0.098
Diabetes mellitus	4 (36%)	11 (23%)	39 (33%)	0.874
Tobacco use	4 (36%)	10 (21%)	33 (28%)	0.730
Family history of CAD	2 (18%)	23 (48%)	51 (43%)	0.047
Prior Angina	5 (45%)	17 (35%)	55 (46%)	0.802
Peripheral arterial disease	1 (9%)	10 (21%)	33 (28%)	0.460
Congestive heart failure	2 (18%)	3 (6%)	9 (8%)	0.958
Myocardial infarction	3 (27%)	11 (23%)	48 (40%)	0.050
Prior CABG	3 (27%)	4 (8%)	22 (18%)	0.104
Prior PCI	3 (27%)	16 (33%)	47 (40%)	0.401
Prior Stroke	0 (0%)	3 (6%)	18 (15%)	0.445
Medications				
Beta-blocker	7 (64%)	27 (56%)	84 (71%)	0.183
ACE inhibitor	3 (27%)	22 (46%)	53 (45%)	0.397
Aspirin	9 (82%)	36 (75%)	102 (86%)	0.678
Insulin	2 (18%)	5 (10%)	13 (11%)	0.652
Oral hypoglycemic	1 (9%)	7 (15%)	16 (14%)	0.869
Calcium channel blocker	2 (18%)	10 (21%)	15 (13%)	0.681
Clopidogrel	4 (36%)	11 (23%)	30 (25%)	0.393
Lipid panel (mmol/L)				
Total cholesterol	160 [134-161]	157 [136-177]	161 [135-200]	0.593
Low density lipoprotein	88 [81-106]	95 [80-112]	92 [74-131]	0.815
High density lipoprotein	43 [37-51]	38 [33-47]	38 [32-44]	0.128
Triglycerides	89 [55-131]	111 [85-199]	132 [100-194]	0.098
Angiographic data				
Obstructive CAD †	10 (91%)	39 (81%)	106 (88%)	0.434
Non-obstructive CAD	1 (9%)	8 (17%)	19 (16%)	0.819
Presence of collaterals	3 (27%)	16 (67%)	42 (60%)	0.868
Cytokines (pg/mL)				
CXCL1	10516 [2094-27454]	4087 [899-18355]	7709 [1749-20804]	0.026
CXCL3	987 [288-1351]	514 [230-1163]	632 [215-1003]	0.359
CXCL5	5820 [4041-15103]	5127 [2326-8607]	5820 [2985-9245]	0.290
CXCL8	741 [517-1391]	751 [367-1441]	758 [345-1664]	0.884
CXCL9	4133 [68-16835]	3421 [297-7749]	3558 [392-7741]	0.890
CXCL10	1552 [922-2075]	922 [587-1362]	901 [587-1670]	0.117
CXCL11	7577 [2920-9914]	5072 [2711-7724]	4449 [2786-7500]	0.259
CXCL12	2316 [2255-11071]	2362 [2016-10622]	2189 [1968-2705]	0.042
CCL2	131 [32-163]	58 [39-92]	62 [28-132]	0.259
VEGF	909 [559-3997]	1219 [205-1991]	957 [147-1880]	0.678
IFN-γ	472 [92-1034]	317 [113-472]	304 [147-525]	0.467

Data are expressed as mean ± standard deviation (SD), median [25-75% interquartile range (IQR)], or as number (percentage), ACE= angiotensin converting-enzyme, CABG= coronary artery bypass graft surgery, CAD= coronary artery disease, IFN= interferon, PCI= percutaneous coronary intervention, VEGF= vascular endothelial growth factor *= on antihypertensive medications, or untreated patients with known systolic blood pressure ≥140mmHg or diastolic blood pressure ≥90mmHg †= ≥70% luminal diameter narrowing of at least one major epicardial artery.

While the angiogenic chemokines, CXCL1 and CXCL12, were significantly different across the groups, there were no differences in median levels of angiostatic chemokines, VEGF or interferon-gamma. Median levels of CXCL1 were highest in subjects not on statin therapy. Compared to subjects either not on statin therapy or on low-dose statins, those taking high-dose statins had lower median values of CXCL12 (2316 [2255–11071], vs 2362 [2016–10622], vs 2189 [1968–2705] pg/mL, p=0.042). CXCL12 levels in those not on statin therapy were similar to those on low-dose statin (2316 [2255–11071] vs 2362 [2016–10622] pg/mL, respectively, p=0.181), suggesting that low-dose statins do not increase CXCL12 levels but rather high-dose statins decrease CXCL12 levels. On multivariate analysis, after adjusting for age, gender, prior myocardial infarction, and chronic administration of aspirin, clopidogrel, insulin, oral hypoglycemic agents, beta-blockers and calcium channel blockers, CXCL12 remained the only factor that was strongly and inversely associated with statin dose at the 95% level (p=0.011). While all subjects had been on statin therapy for at least 2 weeks prior to coronary angiography the duration of statin therapy beyond 2 weeks was not known in all subjects. In the 81 subjects in whom the duration beyond 2 weeks was known, however, there was no difference in the median number of weeks of statin therapy between those on low-dose versus high-dose statin (48 [21–108] vs 49 [25–88], p=0.832).

## Discussion

The effect of statins on angiogenesis is dose-dependent and independent of lipid lowering [2,3]. At low doses, statins promote angiogenesis by enhancing endothelial cell proliferation, migration and differentiation, while at high doses they exert anti-angiogenic effects by inducing endothelial cell apoptosis [2,3]. In addition, statins promote the trafficking of bone marrow-derived progenitor cells into atherosclerotic plaque [7]. In patients with coronary artery disease, statins have been associated with down-regulation of several chemokine ligands [8], but their potential effect on CXCL12, a potent mediator of angiogenesis, has not been reported previously.

CXCL12 plays a unique role in angiogenesis. CXCL12 is constitutively expressed in the bone marrow, and peripheral tissues including the heart, and mediates angiogenesis by regulating the trafficking of endothelial progenitor cells [9]. In a mouse model of pulmonary hypertension, administration of pravastatin ameliorated hypoxia-induced pulmonary hypertension and was associated with decreased accumulation of bone marrow-derived progenitor cells in the pulmonary artery adventitia and reduced plasma levels of CXCL12 [10]. In a hind-limb ischemia model, investigators determined the effects of fluvastatin and CXCL12 on angiogenesis [11]. They found that treatment with fluvastatin and CXCL12 together additively increased migration and proliferation of endothelial progenitor cells into the ischemic issue, improved reperfusion, and down-regulated apoptosis more effectively than treatment with either agent alone [11]. Whether statins play a role in the progression of atherosclerosis was examined in a separate study where apolipoprotein E knockout mice on an atherogenic diet were treated with rosuvastatin (1mg and 10mg/kg of body weight) or pravastatin (10mg/kg of body weight) [7]. They found that treatment with rosuvastatin (particularly in those treated with a high dosage of the statin), but not pravastatin, dose-dependently-reduced the size of atherosclerotic plaques [7]. Moreover, compared to control-treated animals, the plaques in statin-treated animals were characterized by a higher degree of endothelialization and increased numbers of bone marrow-derived progenitor cells within the lesions [7]. Our finding that the angiogenic chemokine CXCL1 was significantly higher in a small number of subjects not on statin therapy is consistent with a previously published paper where investigators found that in 8 subjects with coronary artery disease who were not on statin therapy at baseline, 6 months of high-dose atorvastatin treatment (80mg daily) was associated with a significant decrease in the gene expression of CXCL1 in their peripheral blood mononuclear cells [12]. Lastly, the heterogeneity in blood levels of CXCL12 is consistent with that of other chemokines and has been noted before [5,13]. Our data suggests that statin dose is important and may shed light on the mechanism by which statins exert a biphasic effect on angiogenesis. We show that plasma CXCL12 levels in patients on statin therapy were strongly and inversely associated with statin dose. This finding maybe clinically important in that high doses of statins may inhibit neovascularization in humans which could manifest as stabilization of vulnerable atherosclerotic plaques by suppressing angiogenesis within the plaque, or by inhibiting neovascularization within the ischemic myocardium. Thus, additional human studies with larger numbers of participants are needed to define the effect of statin dose on angiogenesis directly.

Our study has limitations. First, although all subjects were on chronic statin therapy for at least 2 weeks, the duration of statin therapy prior to 2 weeks was not available for all patients. However, in those in whom the duration was known, there was no difference in median number of weeks of statin therapy prior to coronary angiography in those taking low and high-dose statins. Second, the small sample size may have limited our ability to find other differences between the groups. Third, we may have underestimated the extent of collaterals by measuring only spontaneously visible coronary collaterals. Fourth, we did not measure peripheral blood endothelial progenitor cells which may be impacted by CXCL12 levels. Lastly, our results reflect an association but do not establish a causal relationship between statin dose and CXCL12 levels.

## Conclusions

Compared to no therapy or low-dose statin therapy, treatment with high-doses of HMG-CoA reductase inhibitors is associated with decreased circulating CXCL12 levels in subjects with hyperlipidemia, and CXCL12 is strongly and inversely associated with statin dose. Additional studies are needed to confirm this finding in other cohorts and to determine if high-dose statins affect angiogenesis in patients.

## Abbreviations

CAD: Coronary artery disease; IFN: Interferon; VEGF: Vascular endothelial growth factor.

## Competing interests

The authors declare that they have no competing interests.

## Authors’ contributions

WC participated in the design of the study, collected and entered data into the database, helped with the initial statistical analysis, and wrote the first draft of the manuscript. MB helped in the design of the study, carried out the cytokine analyses, and helped to draft the manuscript. RMS participated in the conception of the study, helped to analyze the cytokine data, and helped to draft the manuscript. BM helped in the design of study, directed the cytokine analysis, helped to interpret the results, helped to draft the manuscript. ECK conceived of the study, designed the study, helped with data collection and entry, performed the initial statistical analysis, and helped to draft the manuscript. All authors read and approved the final manuscript.

## Funding

This work was supported by the National Institutes of Health [HL097074 to E.C.K., and HL098526 and HL098329 to B.M.]
